# A Strategy to Setup Codominant Microsatellite Analysis for High-Resolution-Melting-Curve-Analysis (HRM)

**DOI:** 10.1186/1471-2156-9-69

**Published:** 2008-11-03

**Authors:** Eduard Mader, Brigitte Lukas, Johannes Novak

**Affiliations:** 1 Institute for Applied Botany and Pharmacognosy, University of Veterinary Medicine, Veterinärplatz 1, A-1210 Vienna, Austria

## Abstract

**Background:**

High resolution melting curve analysis (HRM) is a technique that measures exactly the decreasing fluorescence of intercalating dye in the process of dissociation of double stranded DNA. The measurement is immediately following PCR in a one-step, closed-tube method. The shape of the melting curve depends on the GC content, length and sequence of the amplicon. Hence it is a powerful, fast and cheap method to detect Single Nucleotide Polymorphisms (SNPs) and other mutations.

**Results:**

Here we present a strategy to set up microsatellite analysis for HRM including the correct assignment of heterozygous samples by comparative analysis and artificial mixtures of samples. The approach is demonstrated on two Simple Sequence Repeat (SSR) loci of different complexity in the genus *Origanum*. Following this strategy all alleles of our sample sets could be classified correctly.

**Conclusion:**

HRM can be used in microsatellite analysis and other codominant marker systems implementing a protocol of comparative melting curve assignment with artificial mixtures of samples to overcome difficulties in correctly assigning heterozygous samples. The method is faster, more sensitive and cheaper than standard protocols for microsatellite analysis.

## Background

High resolution melting curve analysis (HRM) has been introduced several years ago, extending the possibilities of the analysis of DNA melting curves, a standard diagnostic feature in qPCR [1], towards a sensitive method in detecting mutations. The main field of application is the reliable and fast determination of SNPs [2], where the sensitivity of the method has already been widely demonstrated [3]. It is also used for assessment of DNA methylation [4]. HRM also proved applicable for larger sequence aberrations such as Internal Tandem Duplications (ITDs) [5]. It was used for the detection of unknown mutations in a region of low complexity [6] and recently also for analysis of microsatellites [7].

HRM can be seen as an offspring from qPCR technology, where fluorescent dyes are used to detect the quantity of double stranded DNA during the PCR. At the end of the PCR, the fluorescence signal level is high, due to the high amount of dsDNA with intercalated fluorescence dye accumulated in the process. Primer dimers and other non-specific products can subsequently be detected in most qPCR instruments by melting the dsDNA and a stepwise measurement of the decreasing fluorescence. The distance between T_m _values allows the discrimination of the targeted amplicon and non-specific products [1]. Technological progress facilitated a decrease of the size of temperature steps between fluorescence measurement points in the melting process, generating more data and thus drawing a high resolution curve of the level of fluorescence. The HRM curves obtained are highly characteristic for each amplicon and depend on GC content, amplicon length and sequence [8].

Variation in microsatellite loci is often visualized by electrophoresis on polyacrylamide gels, which is principally sensitive only to amplicon-length. Gel-electrophoresis is not only time-consuming, but may also lead to problems in interpretation due to stutter bands. The nowadays common method of microsatellite analysis with multiplex PCR and capillary electrophoresis affords the use of labeled primers, which have to be optmized for multiplex PCR. The problem of stutter peaks also occurs and the method is still quite costly offside routine analysis with well known organisms and markers. Above this it requires at least one additional step following PCR.

Here we describe a strategy to develop a co-dominant microsatellite marker system in diploid organisms using HRM with the advantages of visualization immediately following PCR, low costs and higher sensitivity than electrophoretic detection systems.

Microsatellite primers for *Origanum vulgare*, which have also cross-amplified in other species of the genus *Origanum *[9] are used to demonstrate the strategy.

## Methods

PCR and HRM were performed on a RotorGene 6500 (Corbett Research Pty Ltd, Sydney, Australia) with a HRM-module, the results were analysed using the RotorGene 6000 series software, Version 1.7.65. To obtain interrun comparability standard samples were used in every run.

DNA was extracted from dried leaves of *Origanum *using a modified CTAB extraction protocol [10]. The samples represent a subset from a study on natural hybridizations in the section *Majorana *of the genus *Origanum *(data in preparation). The individuals tested were taken from populations in southern Turkey, sample set 1 consists of 33 individuals of *Origanum onites *(from nearby Manavgat, province of Antalya), sample set 2 comprises 27 individuals of *Origanum majorana *(same location). Sample set 3 is a mixed set of 22 individuals from a total of 11 populations from Southern Turkey (*Origanum onites *and *Origanum majorana*).

The 10 μl PCR reaction volume contained 5.6 μl H_2_O dest., 0.4 Units Taq HOT FIREPol® polymerase, 1 μl Buffer B (Solis BioDyne, Tartu, Estonia), 1.4 μl MgCl2 (25 mM), 0.2 μl DMSO (14.08 M), 0.1 μl dNTP-mix (10 mM), 0.1 μl of each Primer (10 µM) and 0.6 μl fluorescent dye BEBO 36 μM (TATAA Biocenter [11]). 1 μl DNA solution was added to each reaction, containing between 0.25 and 0.8 ng/μl DNA. The mixed samples also contained 1 μl DNA solution with the two compounds at a ratio of 1:1 or 1:2. All reactions were done in duplicate.

The PCR cycling started with an initial phase of 15 min (for the Taq HOT FIREPol^® ^polymerase) at 95°C, then 40 cycles of 10 s at 95°C, 20 s at 60°C and a 20 s elongation step at 72°C. High resolution melting was carried out immediately following PCR from 70°C to 90°C at steps of 0.05°C, each step with a 1 s hold.

For the locus SSR214 the forward primer is 5'-TGTTTGGTGGAAACCGATCC-3', the reverse primer is 5'-AGACGACGAGCTCCAATAACG-3', and the repeat motif is GAT. For the locus SSR244 the forward primer is 5'-TCAAGGGTAGAGCTGCTGCAG-3', the reverse primer is 5'-GCTTTACGGAGGAAGAATGGG-3', and the repeat motif is (GAT)3GAA(GAT)4. [9]

For verification 20 samples were selected for sequencing. Their PCR products were checked on 1.4% agarose gels before being purified with EXO1 and SAP (Fermentas GmbH, St. Leon-Rot, Germany) according to the manufacturer's instructions. Sequencing of both strands was performed using BigDye Terminators (Applied Biosystems, Brunn am Gebirge, Austria) and primers from the original amplifications. The sequences were generated with an ABI 3130x automated sequencer (Applied Biosystems, Brunn am Gebirge, Austria) and edited using Chromas Vers. 2.24 (Technelyseum, Tewantin, Australia).

Observed and expected heterozygosities as well as the fixation index (an index ranging between -1 and +1, values close to zero indicate random mating), were calculated with the software Microsatellite analyzer MSA 4.05 [12].

## Results

### Strategy to setup codominant microsatellite-analysis

In order to demonstrate the usability of HRM for analysing microsatellites, two microsatellites were chosen from a set of recently published sequences [9], one with low complexity (three alleles), and the other more complex with seven alleles in the sample sets.

**Figure 1:**
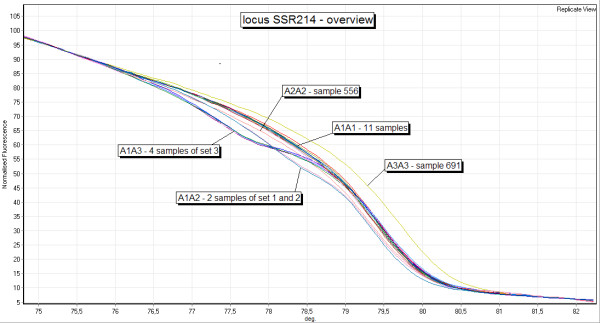
**Overview of all curve forms appearing in locus SSR214**. Melting curves of the homozygous genotypes A1A1, A2A2, A3A3 and of the heterozygous genotypes A1A2 and A1A3. The genotype A2A3 does not occur in the samples analyzed.

**Figure 2:**
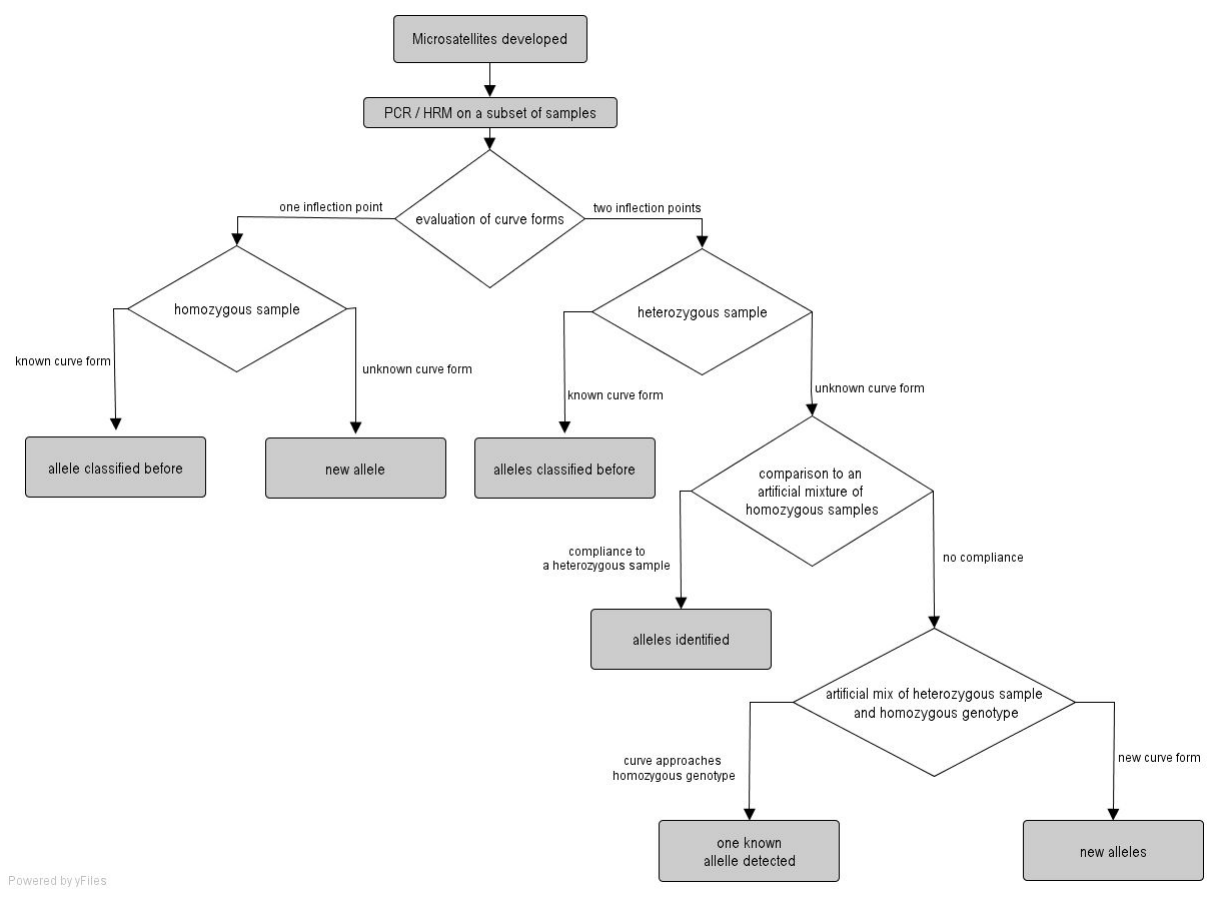
Flowchart outlining the approach to identify the alleles.

At both loci HRM curves showed significant variation, the various curve shapes could be grouped into single inflection point graphs and graphs with two inflection points. Considering the length of the amplicons used (92 bp and 148 bp, respectively), PCR products of homozygous samples should have only one melting domain [13] and thus one inflection point. Consequently an additional melting domain indicates the presence of an additional PCR product. Since *Origanum *is a diploid organism, heterozygous samples should theoretically have two different DNA-strands in a ratio of 1:1 of the amplicons. The PCR products then consist of four different double-stranded DNAs (dsDNAs), A_1_A_1_, A_2_A_2_, A_1_A_2_, A_2_A_1_, the latter two being called heteroduplexes [14]. The heteroduplex dsDNA shows an imperfect binding site of the strands where the additional repetition(s) of the microsatellite occur in one allele. This lowers the melting temperature (T_m_) of the heteroduplexes compared to the related homoduplexes. Therefore, heterozygous samples have two inflection points, and the inflection point at the lower melting temperature is caused by the heteroduplexes, while the second inflection point is caused by homoduplexes. Following this rule, homozygous samples can easily be classified by their curve shape (and between each other by their melting temperature). As mentioned above, interrun comparability was obtained by reference samples of each curve form. Although identical genotypes showed a high congruency (Figure 1), the classification of heterozygous samples by their HRM curve, however, can not be given immediately, because the curve shape does not allow an immediate assignment of the alleles present, as e.g. in size-based electrophoresis. Depending on the alleles present, a comparative identification is nevertheless possible and can be performed according to the flowchart presented in Figure 2.

**Figure 3:**
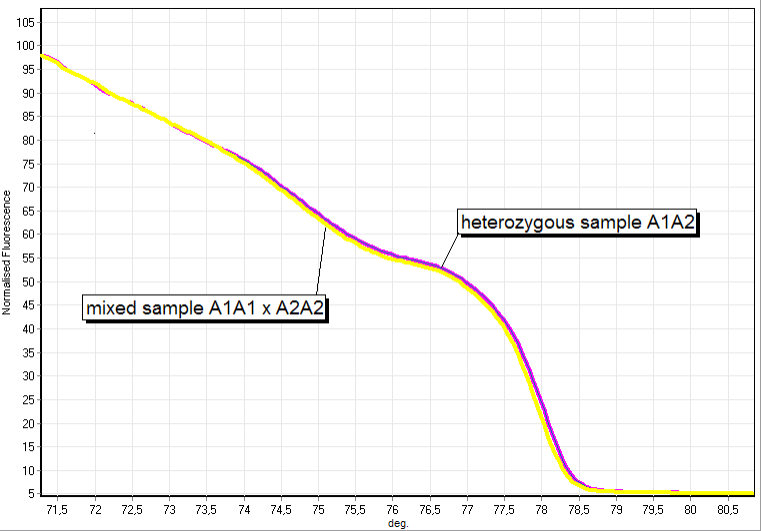
**Comparative identification of heterozygous samples**. Melting curves of a heterozygous sample A1A2 and an artificial mix between the two homozygous samples A1A1 and A2A2 in a ratio of 1:1 at the locus SSR244.

**Figure 4:**
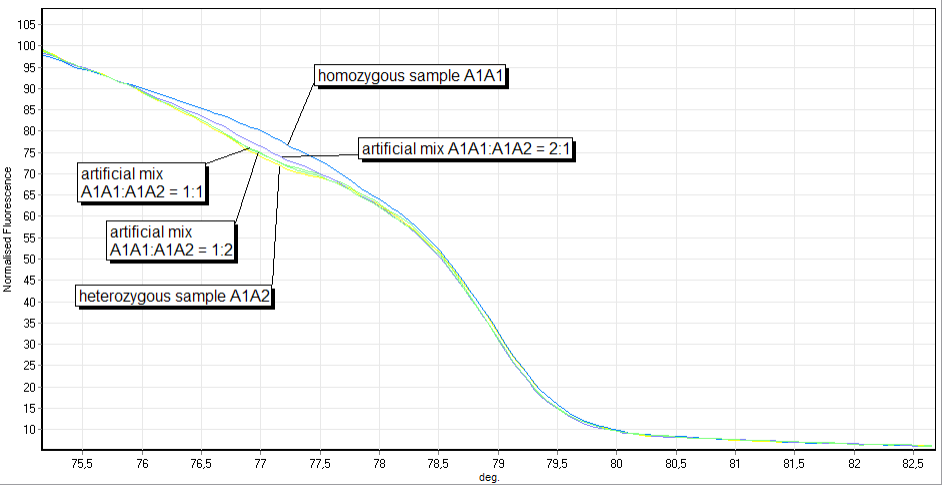
**Artificial mixes of samples at locus SSR214**. Locus SSR214: Melting curves of a heterozygous sample A1A2, a homozygous sample A1A1 and artificial mixes of both in the ratio 1:2, 1:1 and 2:1.

Classification of heterozygous samples is possible based on the fact that artificial sample mixtures of two different homozygous samples will result in exactly the same curve shape like the heterozygous samples consisting of the two alleles of the two homozygous samples in the artificial mixture (Figure 3). If the heterozygous sample curve is not congruent with one of the curves of the artificial homozygous sample mixtures, no homozygous sample was found so far for one or for both allele(s) of the heterozygous sample. In this case, the heterozygous sample can be mixed consecutively with all the homozygous samples. If the curve shape of one of these curves lies exactly between the curve of the heterozygous and the respective homozygous sample, one of the alleles in the heterozygous sample is identical to the allele in the homozygous sample (Figure 4). The reason is that the PCR product of the mixed sample contains then the same dsDNAs like the heterozygous sample, only in a different quantitative composition. Given the other case that the heterozygous sample does not contain the allele of the homozygous sample, the PCR product of the artificial mix will contain two additional heteroduplexes of the allele, the total number of alleles in the mix being three, and the number of different amplicons being six. The presence and comparatively higher ratio of heteroduplexes versus homoduplexes will then lead to a curve shift that differs significantly from the heterozygous sample. Therefore the presence of a known allele in an unidentified heterozygous sample can be determined. If the curves of the artificial heterozygous/homozygous mixtures do not converge to any of the homozygous curves, the heterozygous sample consists of two new alleles.

With this approach the characteristics of the two loci in the sample sets of *Origanum sp*. were elaborated.

### Results of allele analysis

Table 1 gives a summary of the calculated population genetic data. These results are consistent with those of former studies based on gel electrophoresis [9] and therefore confirm the correctness of HRM analysis.

**Table 1:** Results of microsatellite allele analysis

	**Locus**	**N**	**Na**	**Ho**	**He**	**F**
***Sample set 1***	***SSR214***	33	2	0.091	0.088	-0.04
	***SSR244***	33	5	0.364	0.409	0.11

***Sample set 2***	***SSR214***	27	2	0.481	0.439	-0.11
	***SSR244***	27	3	0.556	0.451	-0.25

***Sample set 3***	***SSR214***	22	2	0.136	0.201	0.32
	***SSR244***	22	3	0.364	0.638	0.43

Sample sizes (N), number of alleles (Na), observed (Ho) and expected (He) heterozygosity, and fixation index (F) of the three sample sets

**Figure 5:**
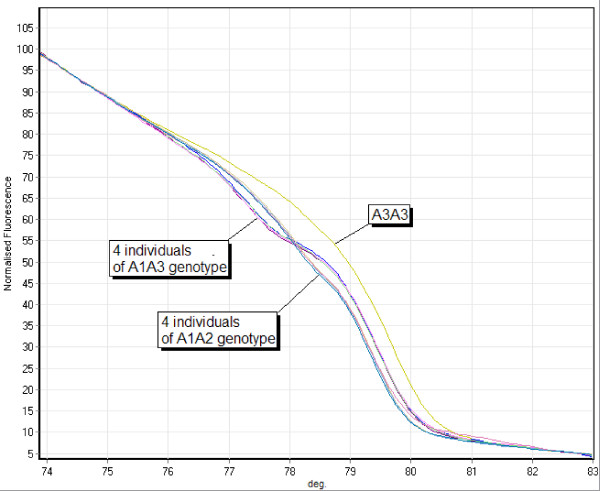
**Precision of detection of various curve shapes and genotypes**. Locus SSR214: Melting curves of one homozygous sample A3A3 and 4 individual samples of A1A3 and A1A2, respectively.

At locus SSR214 all 3 alleles are present in homozygous and heterozygous individuals. Therefore both heterozygous genotypes occurring (A1A2, A1A3) could be classified by comparison to mixed samples of the homozygous individuals. Allele A3 appears in the mixed sample set 3 only. An overview of all curve forms for this locus is given in Figure 1. Figure 5 shows the melting curves of 9 samples of three different genotypes to clarify the precision of HRM analysis. Eight samples representing different curve forms were sequenced. These sequences confirmed HRM analysis. (data not shown)

**Figure 6:**
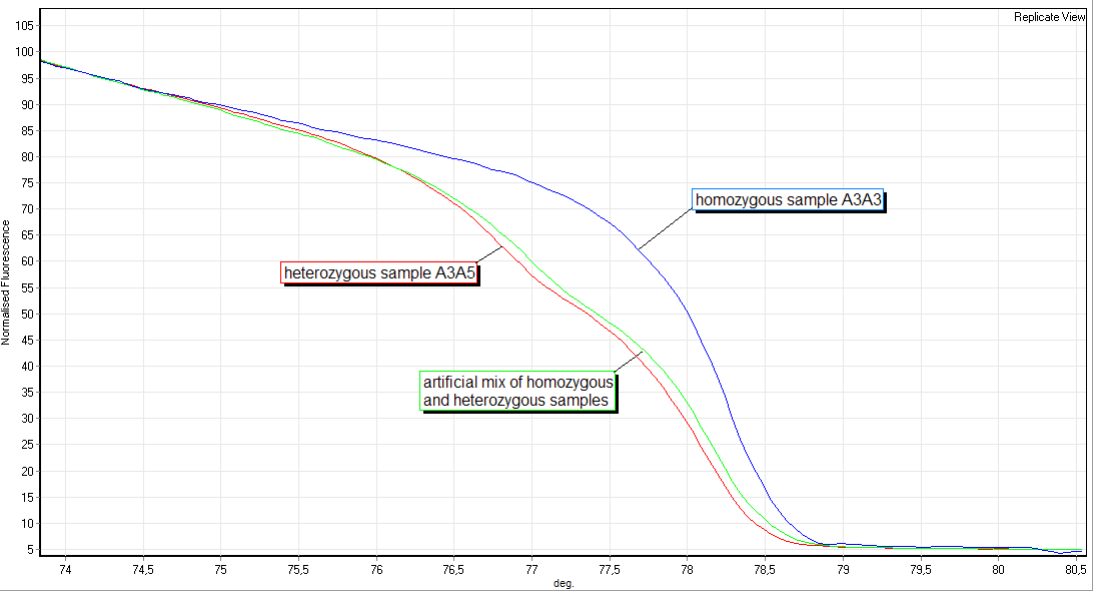
**Artificial mixes of samples at locus SSR244**. Locus SSR244: The HRM curve of an artificial mixture of a homozygous (sample 514, genotype A3A3) and a heterozygous sample (513, genotype A3A5) and the curves of both constituents in the same run.

At locus SSR244 the HRM curves revealed seven different heterozygous genotypes, but only three different homozygous genotypes. Four alleles were found which do not occur in homozygous genotypes in the samples tested. These alleles could be correctly assigned with artificial mixes of the respective heterozygous sample and each homozygous genotype available as shown on an example in Figure 6 at samples of the genotypes A3A3 and A3A5. For verification of HRM results at this locus 12 samples of various curve forms were sequenced and confirmed HRM results.

## Discussion

The experiment demonstrated that the strategy to setup codominant microsatellite analysis worked perfectly well. With known and thoroughly tested microsatellite markers of grapevine HRM was recently implemented by MacKay et al[7]. In species or taxa where no such markers exist yet, like *Origanum*, the lack of certified reference DNA and the uncertainty of alleles present makes an elaborated development strategy for melting curve analysis necessary. To overcome the difficulties with correctly assigning heterozygous samples, the artificial mixes proposed proved to be accurate. The bottleneck in the setup of such a comparative analysis of dissociation curves is the number of different alleles in a given subset of samples. Therefore the sample size to setup this approach has to be increased when the number of heterozygous genotypes is high in order to correctly identify alleles, since the presence of at least some homozygous individuals is necessary to follow this approach. Considering this fact together with the comparative inexpensiveness and ease of use, HRM can be classified as a method with a mid-range capacity. With the ongoing advances in PCR-technology and miniaturization [15], HRM has the capacity to develop into a high throughput method.

Concerning the number of alleles in a population, there is no limit for analysis with HRM as long as a sufficient number of homozygous controls is available to mix with. The only case, which is not entirely resolvable with mixes is the occurrence of two rare alleles in a heterozygous genotype never occurring in a homozygous form. Sequencing such genotypes as well as unexpected genotypes can fill this gap.

As recently shown [16] interrun comparability of melting profiles is also manageable without reference samples in each run, which of course expands the number of samples that can be tested in one run. Furthermore a database utility of melting plots is already envisaged by MacKay et al [7].

To further increase sample throughput, a low number of alleles would allow sample pooling and could enable even higher throughputs with today's standard equipment. For correct classification of pooled sample melting curves it would be necessary to run mixed samples of known genotypes as calibrators or to re-run samples with ambiguous curves individually. A slightly modified strategy could be used to setup the analysis of organisms with higher ploidy levels. Here, quantitative variation between heterozygous and homozygous samples ('curve convergings') could be used to assign the correct allele composition.

HRM also reveals additional mutations in putative microsatellite loci, which can be seen as a great advantage in comparison to electrophoretic analysis, since the alleles of imperfect microsatellites often can not be correctly classified by their length, due to electrophoretic homoplasy of additional SNPs [17]. Additional mutations may also occur quite frequently in the flanking regions of microsatellites. In this aspect melting curve analysis has a great potential in the study of loci involving both SSR and SNPs [18], expanding the observed polymorphisms and thus lead to deeper insights into population structures [19].

## Conclusion

With the implementation of a systematic comparison with artificial mixes of samples – as shown herein – HRM becomes a useful tool for the fast, reliable and cheap analysis of codominant markers. The high sensitivity is a major advantage over electrophoretic analysis when unknown mutations occur. Hence HRM has the potential to substitute other techniques in microsatellite analysis.

## Authors' contributions

EM carried out the experimental work, developed the presented method and drafted the manuscript. BL collected the samples and did experimental preliminary work. JN participated in the design of the experiment and drafted the manuscript. All authors read and approved the final manuscript.
